# Resistin enhances angiogenesis in osteosarcoma via the MAPK signaling pathway

**DOI:** 10.18632/aging.102423

**Published:** 2019-11-13

**Authors:** Hsiao-Chi Tsai, Shih-Ping Cheng, Chien-Kuo Han, Yuan-Li Huang, Shih-Wei Wang, Jie-Jen Lee, Cheng-Ta Lai, Yi-Chin Fong, Chih-Hsin Tang

**Affiliations:** 1 Department of Medical Research, MacKay Memorial Hospital, Taipei, Taiwan; 2 Department of Surgery, MacKay Memorial Hospital and Mackay Medical College, Taipei, Taiwan; 3 Department of Medicine, Mackay Medical College, New Taipei City, Taiwan; 4 Department of Biotechnology, College of Health Science, Asia University, Taichung, Taiwan; 5 Graduate Institute of Natural Products, College of Pharmacy, Kaohsiung Medical University, Kaohsiung, Taiwan; 6 Division of Colon and Rectal Surgery, Department of Surgery, MacKay Memorial Hospital, Taipei, Taiwan; 7 Department of Sports Medicine, College of Health Care, China Medical University, Taichung, Taiwan; 8 Department of Orthopedic Surgery, China Medical University Hospital, Taichung, Taiwan; 9 School of Medicine, China Medical University, Taichung, Taiwan; 10 Chinese Medicine Research Center, China Medical University, Taichung, Taiwan

**Keywords:** resistin, VEGF-A, MAPK, angiogenesis, osteosarcoma

## Abstract

Over the last two decades, there have been no significant changes in patient outcomes in relation to the treatment of osteosarcoma, an aggressive malignant neoplasm. It is known that vascular endothelial growth factor-A (VEGF-A) plays a crucial role in angiogenesis and in osteosarcoma. Moreover, VEGF-A expression correlates with clinical stages of osteosarcoma. The adipokine resistin exhibits proinflammatory, proangiogenic and metastatic properties, and evidence suggests that resistin may serve as a prognostic biomarker linking obesity and inflammation to cancer. However, whether resistin has a role in osteosarcoma angiogenesis is unclear. This investigation shows that resistin promotes VEGF-A expression in human osteosarcoma cells and activates the extracellular signal-regulated kinase (ERK), c-Jun N-terminal kinase (JNK) and p38 signaling pathways, while ERK, JNK, and p38 inhibitors or their small interfering RNAs (siRNAs) inhibit resistin-induced VEGF-A expression as well as endothelial progenitor cell (EPC) migration and tube formation. We also found that resistin upregulates VEGF-A expression by enhancing activation of the transcription factor nuclear factor-kappa B (NF-κB). Finally, resistin promotes angiogenesis in the chick chorioallantoic membrane (CAM) model. Resistin appears to be a promising target for human osteosarcoma.

## INTRODUCTION

Osteosarcoma accounts for 30–80% of primary skeletal sarcomas and is well-recognized for its high metastatic rates, which are believed to underline the poor survival rates in this disease. Lung metastases are found in approximately 20% of patients at initial diagnosis; around 40% of patients develop metastases at a later stage [1]. Following the development of metastases, osteosarcoma has a 5-year survival rate of 20% [2]. Angiogenesis is key to the development of osteosarcoma, its subsequent progression and metastasis [3, 4]. Results from using differential co-expression network, finding angiogenesis is closely related to osteosarcoma progression and metastasis [5]. Thus, antiangiogenic therapy might be feasible for osteosarcoma patients [6].

Vascular endothelial growth factor A (VEGF-A; also referred to as VEGF) is a major promoter of angiogenesis [7]. Antiangiogenic inhibitors targeting the VEGF-A pathway are currently used in antitumor therapy [8]. We have previously reported correlations between levels of VEGF-A expression and tumor stages in human osteosarcoma tissue specimens [9, 10]. More clarification is needed as to the effects of VEGF-A in angiogenesis pathways and its underlying mechanisms in human osteosarcoma.

Resistin, an adipocyte-derived cytokine, has been investigated for its role in inflammation and obesity-related cancers [11]. Increasing serum resistin levels correlate with the progression of breast, colon and endometrial cancers [12]. However, the role of resistin in osteosarcoma is unclear. In this study, we examined how resistin affects VEGF-A expression and tumor angiogenesis. We then investigated the signaling pathways underlying resistin-induced VEGF-A-dependent angiogenesis in human osteosarcoma cells. We consider that our findings will be helpful in the development of new treatment strategies for osteosarcoma.

## RESULTS

### Clinical significance of resistin and VEGF-A expression in human osteosarcoma tissue specimens

**Figure 1:**
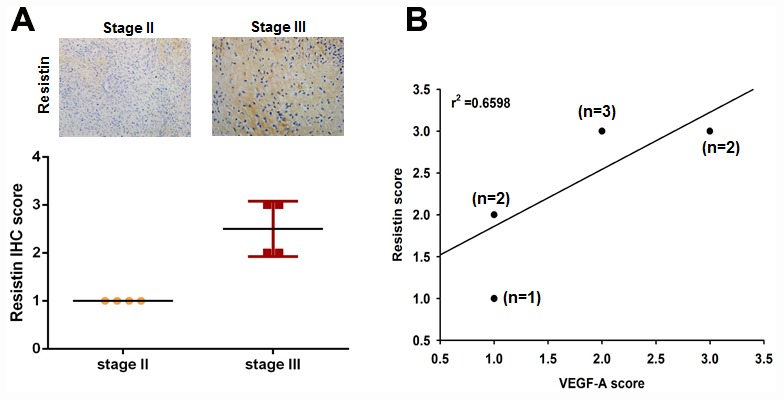
**Clinical significance of resistin expression in tissue specimens from patients with osteosarcoma.** (**A**) Tumor specimens were subjected to immunohistochemistry (IHC) evaluations with anti-resistin monoclonal antibody and the staining intensity was graded as 0 (no positive cell staining), 1 (1–24%, weakly positive), 2 (25–49%, moderately positive), or 3 (50–100%, strongly positive). (**B**) Positive correlations were observed between levels of resistin and VEGF-A expression.

Previous studies have reported that resistin stimulates the growth of many tumors by promoting the secretion of VEGF-A, which promotes tumor invasion [13–16]. However, there are few studies of resistin in relation to osteosarcoma. When we examined resistin expression profiles in human osteosarcoma tissue specimens, we found that higher resistin expression significantly correlated with increasing tumor stage (Figure 1A). It has been reported that circulating VEGF-A levels are associated with the development of lung metastasis and with the clinical outcomes of osteosarcoma [4]. We therefore compared resistin IHC scores with the VEGF-A profiles we had previously obtained from clinical osteosarcoma tissue samples [9, 10] and found a highly positive relationship between resistin and VEGF-A expression (Figure 1B). These results implicate the involvement of resistin in the levels of VEGF-A expression and tumor progression in osteosarcoma patients.

### Resistin regulates angiogenesis by upregulating VEGF-A expression in osteosarcoma cells

**Figure 2:**
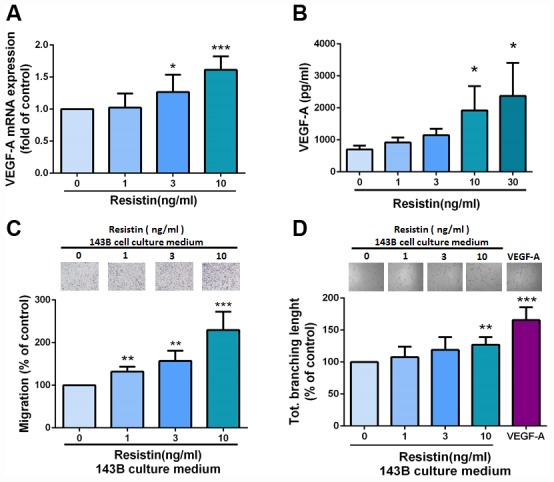
**Resistin mediates angiogenesis by inducing increases in VEGF-A expression in osteosarcoma cells.** (**A**–**B**) After incubating osteosarcoma 143B cells with resistin (0–10 ng/ml) for 24 h, VEGF-A expression was measured by qPCR and ELISA. (**C**–**D**) Osteosarcoma 143B cells were pretreated with resistin (1–10 ng/ml) for 24 h. CM was collected and applied to EPCs for 24 h. Cell migration and capillary-like structure formation in EPCs were examined by Transwell and tube formation assays, respectively. The results were obtained from three independent experiments. * *p* < 0.05; ** *p* < 0.01; *** *p* < 0.001 compared with controls.

As our IHC results indicated that levels of resistin expression correlate with VEGF-A expression in human osteosarcoma specimens, we then sought to determine whether resistin promotes VEGF-A expression in human osteosarcoma. Our results showed that resistin increased levels of VEGF-A expression and secretion in osteosarcoma cells (Figure 2A and 2B). It has been reported that EPCs contribute to neovascularization in tumors [17]. In this study, CM from resistin-treated osteosarcoma cells enhanced EPC migration and tube formation (VEGF-A served as the positive control) (Figure 2C and 2D), indicating that EPCs may be recruited to the tumor microenvironment and form new blood vessels via resistin-promoted VEGF-A expression. This suggests that resistin induces angiogenesis in a VEGF-A-dependent manner.

### The MAPK signaling pathway is involved in resistin-promoted VEGF-A expression and contributes to angiogenesis

**Figure 3:**
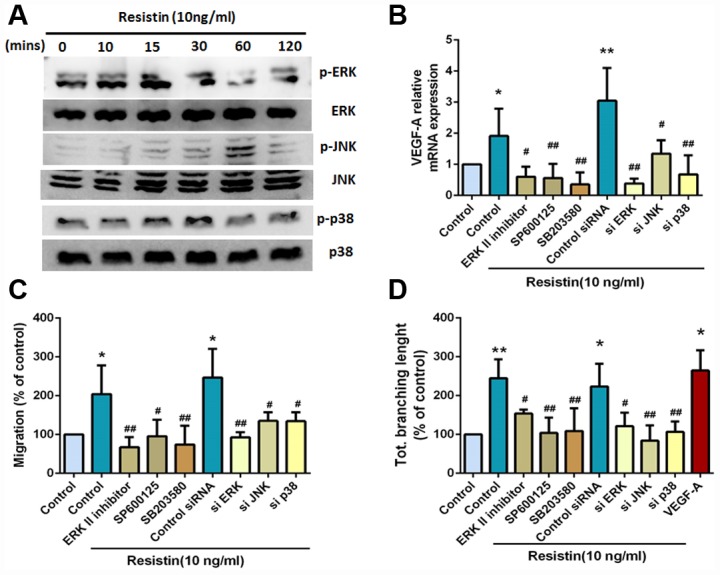
**The MAPK signaling pathway is involved in resistin-promoted VEGF-A expression and contributes to angiogenesis.** (**A**) Osteosarcoma 143B cells were incubated with resistin (10 ng/ml) for the indicated times, and ERK, JNK and p38 phosphorylation was determined by Western blot analysis. (**B**) Osteosarcoma 143B cells were pretreated with an ERKII inhibitor (10 μM), a JNK inhibitor (SP600125; 10 μM) and a p38 inhibitor (SB203580; 10 μM) for 30 min or transfected with ERK, JNK, and p38 siRNAs for 24 h, followed by resistin (10 ng/ml) stimulation for 24 h. VEGF-A expression was examined by qPCR. (**C**–**D**) Osteosarcoma 143B cells were pretreated with an ERKII inhibitor (10 μM), a JNK inhibitor (SP600125; 10 μM) and a p38 inhibitor (SB203580; 10 μM) for 30 min, or transfected with ERK, JNK and p38 siRNAs for 24 h, then stimulated with resistin (10 ng/ml) for 24 h. CM was collected and then applied to EPCs for 24 h. Cell migration and capillary-like structure formation in EPCs were examined by Transwell and tube formation assays, respectively. The results were obtained from three independent experiments. * *p* < 0.05; ** *p* < 0.01; *** *p* < 0.001 compared with controls. ^#^ *p* < 0.05; ^##^ *p* < 0.01 compared with resistin-treated control groups.

Previous research has reported that intracellular signaling of resistin converges in the activation of the mitogen-activated protein kinase (MAPK) signaling pathway [18], which consists of the ERK, JNK and p38 families; their activation is also involved in the regulation of VEGF-A expression [10, 19]. To investigate whether the MAPK pathway affects resistin-induced VEGF-A expression, we incubated cells with resistin and observed subsequent increases in ERK, JNK and p38 phosphorylation (Figure 3A). Pretreatment with inhibitors of ERK, JNK and p38 or their siRNAs reversed resistin-induced increases in VEGF-A expression (Figure 3B). Furthermore, EPC migration and tube formation, which were induced by treatment with the indicated inhibitors or siRNA-treated CM, was also abolished (Figure 3C and 3D). These results indicate that resistin promotes VEGF-A expression in osteosarcoma cells and contributes to angiogenesis by activating the ERK, JNK and p38 pathways.

### Resistin promotes VEGF-A expression in osteosarcoma and contributes to angiogenesis through the NF-κB signaling pathway

**Figure 4:**
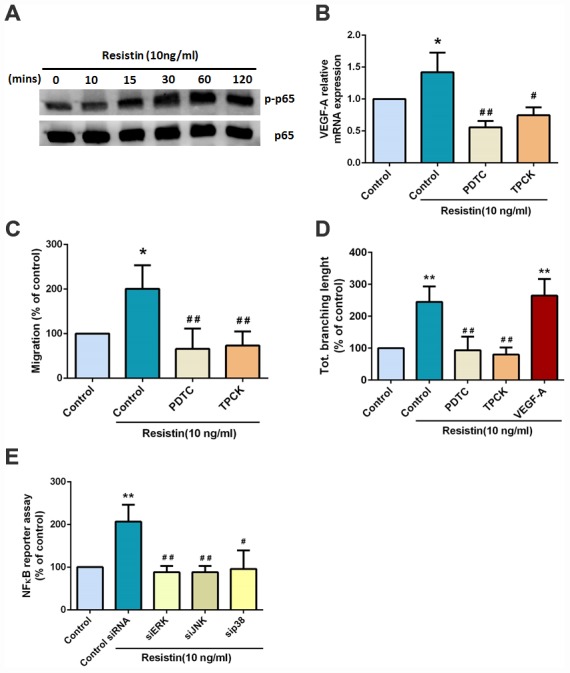
**Resistin promotes VEGF-A expression in osteosarcoma and contributes to angiogenesis through the NF-κB signaling pathway.** (**A**) Osteosarcoma 143B cells were incubated with resistin (10 ng/ml) for the indicated times and p65 phosphorylation was determined by Western blot analysis. (**B**) Osteosarcoma 143B cells were pretreated with NF-κB inhibitors (PDTC 10 μM; TPCK 3 μM) for 30 min then stimulated with resistin (10 ng/ml) for 24 h. VEGF-A expression was examined by qPCR. (**C**–**D**) Osteosarcoma 143B cells were pretreated with NF-κB inhibitors (PDTC 10 μM; TPCK 3 μM) for 30 min then stimulated with resistin (10 ng/ml) for 24 h. CM was collected and applied to EPCs for 24 h. Cell migration and capillary-like structure formation in EPCs were examined by Transwell and tube formation assays, respectively. (**E**) Cells were transfected with indicated siRNA, the luciferase activity was examined. The results were obtained from three independent experiments. * *p* < 0.05; ** *p* < 0.01; *** *p* < 0.001 compared with controls. ^#^ *p* < 0.05; ^##^ *p* < 0.01 compared with resistin-treated control groups.

The transcription factor nuclear factor kappa-B (NF-κB) is involved in VEGF-A expression [20, 21]. In this study, we observed that resistin treatment time- dependently increased NF-κB p65 protein phosphorylation (Figure 4A) (Supplementary Figure 1). Moreover, pretreatment with NF-κB inhibitors PDTC and TPCK reversed resistin-induced increases in VEGF-A expression, EPC migration and tube formation (Figure 4B–4D). In addition, co-transfecting the κB-luciferase plasmid with ERK, JNK and p38 siRNAs also reduced resistin-induced κB-luciferase activity (Figure 4E). These data suggest that resistin regulates VEGF-A expression and promotes angiogenesis via NF-kB transcription factor activation.

### Effects of resistin on VEGF-A-induced angiogenesis in the CAM model

**Figure 5:**
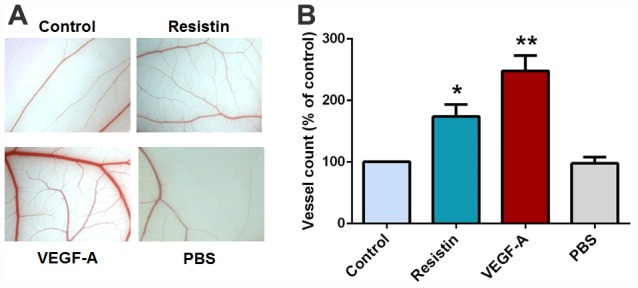
**Effects of resistin on VEGF-A-induced angiogenesis in the CAM model.** (**A**) PBS, VEGF-A (50 ng/ml), control osteosarcoma 143B cell CM and resistin-treated osteosarcoma 143B CM were mixed in Matrigel and subjected to the CAM assay, then photographed with a stereomicroscope on developmental day 12. (**B**) Angiogenesis was quantified by counting the number of blood vessel branches. * *p* < 0.05; ** *p* < 0.01; *** *p* < 0.001 compared with controls.

Finally, we used the *in vivo CAM model to confirm the* effects of resistin on VEGF-A-induced angiogenesis. As expected, CM collected from resistin-treated cells enhanced CAM angiogenesis (VEGF-A served as the positive control) (Figure 5). These results indicate that resistin promotes angiogenesis *in vivo*.

## DISCUSSION

For many tumors, the ability to progress to a state of malignancy depends on the microenvironment. Blood capillaries are usually static under physiological conditions but are induced to proliferate by direct exposure to angiogenic factors, such as VEGF-A [6], which support tumor growth and metastasis [22]. Increasing evidence shows that adipocytes and adipokines are involved in primary inflammatory processes and disease [23]. Resistin, an adipocyte- derived cytokine, has been studied extensively for its role in inflammation and obesity-related cancers [11]. Serum levels of resistin are known to increase in breast, colon, and endometrial cancer during tumor progression [12], although few studies have investigated the role of resistin in bone cancers, especially in osteosarcoma [24, 25]. Evidence has linked increasing resistin expression with poor prognosis and the development of lung adenocarcinoma [26]. Similarly, in our study, IHC results from clinical osteosarcoma specimens showed that resistin and VEGF-A expression levels were positively correlated with increasing tumor stage.

**Figure 6:**
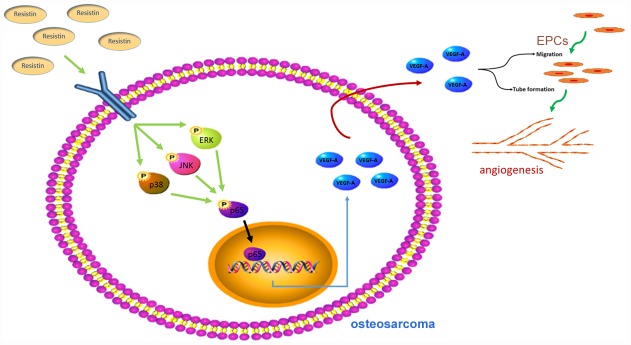
**Schematic presentation of the signaling pathways involved in resistin-induced angiogenesis in human osteosarcoma cells.**

Previous research has reported that intracellular signaling of resistin converges in the activation of the MAPK signaling pathway [17]. The MAPK pathway plays an important role in the regulation of VEGF-A expression levels [9, 24]. Our analyses revealed that resistin time-dependently stimulates VEGF-A production in human osteosarcoma cells via the ERK, JNK and p38 pathway, which subsequently increased EPC migration, tube formation and tumor angiogenesis (Figure 6). Pretreatment of cells with ERK, JNK, or p38 inhibitors prevented resistin-induced increases in VEGF-A expression, EPC migration and tube formation. Our results clearly indicate that the ERK, JNK and p38 pathway enhances resistin-induced stimulation of angiogenic functions by upregulating VEGF-A expression.

In addition to intracellular signaling, levels of oxidative stress are critical in osteosarcoma pathology [27]. Whereas low levels of oxidative stress promote survival and proliferation of cancer cells, higher levels induce apoptosis and cell cycle arrest [28]. Mitochondrial defects have also been reported in many different cancers, including osteosarcoma, and have been considered as a prognostic biomarker [29]. Apoptosis appears to be linked to higher mitochondrial oxidative stress [30]. However, this study failed to detect levels of oxidative stress and subunits of oxidative phosphorylation (mitochondrial markers Complexes I–V). These should be investigated in future research.

In conclusion, resistin is expressed at high levels in osteosarcoma and correlates with clinical stages of this disease. Resistin promotes VEGF-A expression by activating the MAPK signaling pathway, which subsequently promotes angiogenesis. Thus, resistin may represent a new molecular therapeutic target in osteosarcoma angiogenesis and metastasis.

## MATERIALS AND METHODS

### Materials

Human recombinant resistin and VEGF-A protein were purchased from PeproTech (Rocky Hill, NJ, USA). VEGF-A antibody was purchased from Abcam (Cambridge, MA, USA). Antibodies for resistin, ERK, JNK, p38 and p65 (all mouse monoclonal IgG antibodies) and for pERK, pJNK, pp38 and pp65 (all rabbit monoclonal IgG antibodies) were purchased from Santa Cruz Biotechnology (Santa Cruz, CA, USA). SP600125, SB203580, PDTC and TPCK were purchased from Calbiochem (San Diego, CA, USA). ON-TARGETplus small interfering RNAs (siRNAs) of JNK, ERK and p38 were purchased from Dharmacon Research (Lafayette, CO, USA). Lipofectamine 2000 was purchased from Invitrogen (Carlsbad, CA, USA). Reporter lysis buffer was purchased from Promega (Madison, WI, USA) and human osteosarcoma tissue arrays were purchased from Biomax (Rockville, MD, USA). All other chemicals were purchased from Sigma-Aldrich (St. Louis, MO, USA).

### Cell culture

The human osteosarcoma cell line 143B was purchased from the Bioresource Collection and Research Center (BCRC) (Hsinchu, Taiwan). Cells were maintained in Dulbecco’s modified Eagle’s medium (DMEM) supplemented with 20 mM HEPES and 10% heat-inactivated fetal bovine serum (FBS), 2 mM glutamine, penicillin (100 U/ml) and streptomycin (100 μg/ml) at 37°C with 5% CO_2_.

Endothelial progenitor cell (EPC) was purchased from Lonza (Walkersville, MD, USA) and was cultured in MV2 medium that contained MV2 basal medium and growth supplement (PromoCell, Heidelberg, Germany) with the addition of 20% FBS (HyClone, Logan, UT, USA). Cultures were seeded onto 1% gelatin-coated plasticware and maintained at 37°C in a humidified 5% CO_2_ atmosphere.

### Immunohistochemical staining

An osteosarcoma tissue array was deparaffinized with xylene and rehydrated with ethanol. Endogenous peroxidase activity was blocked with 3% hydrogen peroxide in methanol for 10 min. Heat-induced antigen retrieval was carried out for all sections in 0.01 M sodium citrate buffer (pH 6) at 95°C for 25 min. Human resistin antibody was applied at a dilution of 1:250 and incubated at 4°C overnight. The antibody-binding signal was detected using the NovoLink Polymer Detection System (Leica Microsystems) and reaction products were visualized using diaminobenzidine. The sections were counterstained with hematoxylin. Immunohistochemistry (IHC) results were scored by accounting for the percentage of positive detection and intensity of the staining in calculations using MacBiophotonics Image J software. Staining intensity was graded as 0 (no positive cell staining), 1 (1–24%, weakly positive), 2 (25–49%, moderately positive), or 3 (50–100%, strongly positive).

### Reverse-transcribed quantitative real-time PCR

Total RNA was extracted from osteosarcoma cells using a TRIzol kit (MDBio Inc., Taipei, Taiwan). Total RNA (2 μg) was reverse-transcribed (RT) into complementary DNA (cDNA) using an oligo(dT) primer. Quantitative real-time polymerase chain reaction (q-PCR) analysis was carried out using TaqMan^®^ one-step PCR Master Mix (Applied Biosystems, Foster City, CA, USA). Total cDNA (100 ng/25μl reaction) was mixed with sequence-specific primers and TaqMan^®^ probes according to the manufacturer’s instructions. All target gene primers and probes were purchased commercially (GAPDH was used as the internal control) (Applied Biosystems, Foster City, CA, USA). RT-qPCR assays were carried out in triplicate using a StepOnePlus sequence detection system. The cycling conditions were 10 min of polymerase activation at 95°C, followed by 40 cycles at 95°C for 15 s and 60°C for 60 s.

### ELISA assay

Human osteosarcoma cells were cultured in 6-well plates. After reaching confluence, cells were changed to serum-free medium. The cells were then treated with resistin for 24 h. After treatment, the medium was removed and stored at −80°C. The amount of VEGF-A in the medium was determined using a VEGF-A ELISA kit (PeproTech, Rocky Hill, NJ, USA), according to the manufacturer's protocol.

### EPC migration assay

A cell migration assay was performed using 6.5 mm Transwell chambers with 8.0 μm pore polycarbonate membrane sizes (Corning Incorporated, Corning, NY, USA). EPCs (1 × 10^4^ cells/well) were seeded onto the upper chambers with MV2 medium, then incubated in the lower chambers with 50% MV2 medium and 50% osteosarcoma cell culture medium (CM). The plates were incubated for 24 h at 37°C in 5% CO_2_, then the cells were fixed in 4% formaldehyde solution for 15 min and stained with 0.05% crystal violet in phosphate-buffered saline (PBS) for 15 min. Cells on the upper sides of the filters were removed with cotton-tipped swabs and the filters were washed with PBS. Cell migration was quantified by counting the number of stained cells in 10 random fields using an inverted phase contrast microscope and then photographed.

### Tube formation assay

Matrigel (BD Biosciences, Bedford, MA, USA) was dissolved at 4°C overnight and 48-well plates were prepared with 150 μl Matrigel in each well, then incubated at 37°C for 30 min. After gel formation, EPCs (1×10^4^ cells) were seeded into each well onto a layer of polymerized Matrigel in CM containing 50% MV2 complete medium and 50% osteosarcoma cell CM, then incubated for 4 h at 37°C. EPC tube formation was examined using an inverted phase contrast microscope. The number of tube branches and total tube length were calculated using the MacBiophotonics Image J software.

### Western blot analysis

Total protein concentration was determined using the Thermo Scientific Pierce BCA Protein Assay Kit (Thermo Fisher Scientific Inc., Rockford, IL, USA). Proteins were resolved with SDS-PAGE and transferred to Immobilon^®^ polyvinylidene difluoride (PVDF) membranes. The blots were blocked with 5% BSA for 1 h at room temperature then incubated with primary antibodies for 1 h at room temperature. After 3 washes in Tris-buffered saline with 0.05% Tween 20 (TBS-Tween), the blots were subsequently incubated with a donkey anti-rabbit or anti-mouse peroxidase-conjugated secondary antibody for 1 h at room temperature. The blots were visualized by enhanced chemiluminescence using Kodak X-OMAT LS film (Eastman Kodak, Rochester, NY, USA).

### Transfection and reporter gene assay

Osteosarcoma 143B cells were co-transfected with 1 mg κB-luciferase plasmid and 0.5 mg β-galactosidase expression vector, then were grown to 80% confluence in 24-well plates and transfected on the following day with Lipofectamine^®^ 2000 (LF2000; Invitrogen, Carlsbad, CA, USA). DNA and LF2000 were premixed for 20 min before being applied to cells. After 24 h of transfection, the cells were incubated with the indicated agents. After a further 24 h of incubation, the media were removed and the cells were washed once with cold PBS. To prepare lysates, 100 ml reporter lysis buffer (Promega, Madison, WI, USA) was added to each well and the cells were scraped from the dishes. The supernatant was collected after centrifugation at 13,000 rpm for 5 min. Aliquots of cell lysates (20 ml) containing equal amounts of protein (20–30 mg) were placed into wells of black opaque 96-well microplate. An equal volume of luciferase substrate was added to all samples, and luminescence was measured in a microplate luminometer. The value of luciferase activity was normalized using the co-transfected β-galactosidase expression vector as the internal control.

### Chick chorioallantoic membrane (CAM) assay

The influence of resistin on blood vessel formation was performed by a CAM assay using fertilized chicken eggs. Eggs were incubated at 38°C in an 80–90% humidified atmosphere. On day 7, an ostiole of approximately 1 cm^2^ was opened in the air sac of each egg, before adding 100 μl of CM collected from different conditions. CAM results were analyzed on the fourth day. Chorioallantoic membranes were collected for microscopy and photographic documentation. Angiogenesis was quantified by counting the number of blood vessel branches; at least 10 viable embryos were tested for each treatment. All animal investigations were performed in accordance with a protocol approved by China Medical University’s Institutional Animal Care and Use Committee (Taichung, Taiwan).

### Statistics

Statistical data were analyzed using SPSS Statistics version 20.0 (SPSS, Chicago, USA). All data are expressed as the mean ± standard error of the mean. Statistical comparisons between two samples were performed using the Student's *t*-test, and a one-way analysis of variance (ANOVA) with *post hoc* Bonferroni correction was used to compare multiple groups. A value of *p* < 0.05 was considered significant.

## Supplementary Material

Supplementary Figure 1
