# Supplementary material for: Resistin enhances angiogenesis in osteosarcoma via the MAPK signaling pathway
Source: Aging (Albany NY). 2019 Nov 13;11(21):9767–77. doi: 10.18632/aging.102423 (PMC6874472; doi:10.18632/aging.102423)
Supplement: Supplementary Figure 1 [file aging-11-102423-s001.pdf]

SUPPLEMENTARY FIGURE

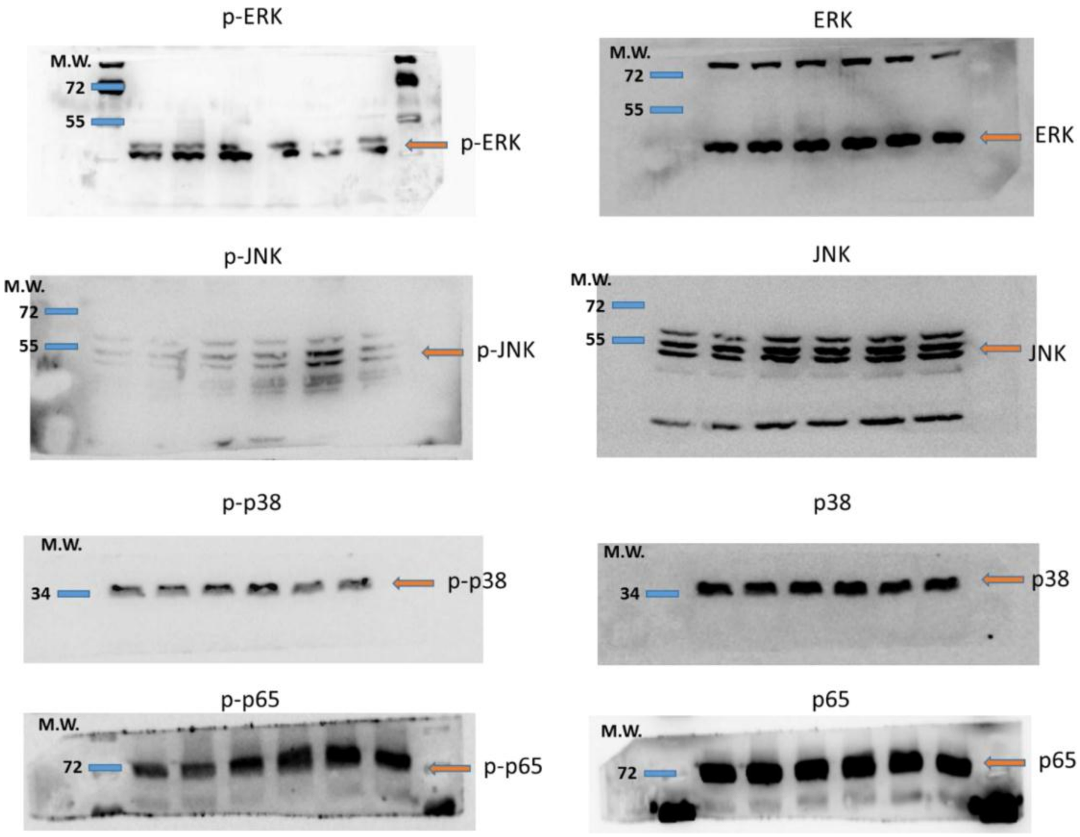

Supplementary Figure 1. The full length of the original immunoblotting results in this study.
